# Physical Activity in High-Risk Pregnancies

**DOI:** 10.3390/jcm11030703

**Published:** 2022-01-28

**Authors:** Christina Sitzberger, Juliane Hansl, Ricardo Felberbaum, Anke Brössner, Renate Oberhoffer-Fritz, Annette Wacker-Gussmann

**Affiliations:** 1 Institute of Preventive Pediatrics, Faculty of Sport and Health Sciences, Technical University of Munich, 80992 Munich, Germany; jhansl@gmx.de (J.H.); renate.oberhoffer@tum.de (R.O.-F.); annette.wacker-gussmann@tum.de (A.W.-G.); 2 Klinikverbund Kempten(Allgäu), Kinderwunschzentrum, 87439 Kempten (Allgäu), Germany; Ricardo.Felberbaum@klinikverbund-allgaeu.de (R.F.); Anke.Broessner@klinikverbund-allgaeu.de (A.B.); 3 German Heart Centre, Department of Paediatric Cardiology and Congenital Heart Defects, 80636 Munich, Germany

**Keywords:** assisted reproductive technology, congenital heart disease, pregnancy, fetal, physical activity

## Abstract

It is known that physical activity before and during pregnancy is associated with health benefits for both the mother and fetus. The WHO recommends a minimum of 150 min per week of moderate-intensity aerobic physical activity for pregnant women. However, the majority of pregnant woman seem not to be physically active in pregnancy as recommended. In addition, the WHO recommendations do not include information on physical activity (PA) for specific target groups. This might be particularly problematic in women with assisted reproduction technologies (ART) or those who have received the fetal diagnosis of congenital heart defects (CHD). The aim of our study was to elaborate on whether assisted reproduction technologies (ART) and/or the diagnosis of fetal congenital heart defects (CHD) influence the level of PA in pregnant women, and to determine if there is a difference between PA behavior before and during pregnancy. In addition, we will evaluate whether high-risk pregnant women also reach the WHO recommendations. A non-interventional, cross-sectional, monocentric study based on two standardized questionnaires on physical activity was conducted. In total, *n* = 158 pregnant women were included. All of the participants were recruited from the outpatient clinics of the German Heart Center, Munich, and the Klinikverbund Kempten-Oberallgäu, Germany. Pregnant women after ART (*n* = 18), with fetal CHD (*n* = 25) and with both ART and CHD (*n* = 8) could be included. A total of 107 pregnant women served as healthy controls. Women, after ART, showed a significantly reduced level of physical activity (*p* = 0.014) during pregnancy compared to women who became pregnant naturally. Additionally, less (*p* < 0.001) and lighter (*p* = 0.002) physical activity was observed in all groups during pregnancy compared to those before pregnancy. An increase in maternal age increases the likelihood of CHD (*p* < 0.001) and decreases the level of physical activity before pregnancy (*p* = 0.012). The overall level of physical activity decreased in healthy and high-risk pregnancies, and only a quarter (26.49%) of all pregnant women reached the WHO recommendations. Further research for the specific target groups is highly recommended in order to promote and increase physical activity in ART and CHD pregnancies.

## 1. Introduction

The World Health Organization (WHO) updated their guidelines on physical activity in the year 2020. While the guidelines had previously only addressed children, adolescents and older adults, recommendations for specific populations including pregnant and postpartum women were implemented in the updated guidelines. Moreover, the updated WHO guidelines provide information on how sedentary behavior negatively influences health and how it can be reduced. It is recommended that pregnant and postpartum women engage in a minimum of 150 min of moderate-intensity aerobic physical activity throughout the week, including a variety of aerobic and muscle-strengthening activities, for instance, running, cycling or swimming. In addition, it is advised that the amount of time spent sedentary be reduced, by replacing it with physical activity of any intensity [1,2]. Research has proven that physical activity before and during pregnancy is associated with several health benefits for the mother as well as for the fetus [2,3,4,5,6,7]. Barker and colleagues suggest several environmental determinants that modify in utero fetal development, causing alterations in metabolism and in vulnerability to chronic diseases in adulthood [8]. Among others, physical activity represents one of the mentioned determinants. Research confirms that engaging in regular physical activity during pregnancy can improve the fetus’ environment and the overall health of the offspring later in life [9]. Despite these findings and the beneficial effects of physical activity during pregnancy, the majority of pregnant women are not as physically active as recommended by the WHO [10,11].

However, the WHO guidelines do not include information on physical activity for pregnancies that deviate from the norm. One could assume that the specified target groups are unsure about whether to follow the guidelines and engage in physical activity before and during pregnancy. It is therefore necessary to make recommendations for these target groups, to improve the health of mothers and their children here as well.

The aim of this study is to bridge the existing research gap by examining whether ART and the diagnosis of fetal CHD have a possible impact on the level of physical activity in pregnant women.

## 2. Materials and Methods

Pregnant women after assisted reproduction technologies (ART) and with the diagnosis of congenital heart defects (CHD) were included in this non-interventional, cross-sectional, monocentric study between August 2015 and May 2021. Healthy pregnant women served as controls. All of the participants (ART and CHD) were recruited at the German Heart Centre, Munich, and at Klinikverbund Kempten-Oberallgäu, Germany. Recruitment was carried out by the research team. The pregnant women were addressed during a fetal consultation hour or existing files via telephone. The suitability for this study, with regard to the inclusion and exclusion criteria, was checked in advance on the basis of the existing files.

Inclusion criteria were pregnancies after ART, the diagnosis of fetal CHD, or both, and the women needed to have reached at least the second trimester. An exclusion criterion was if women did not have sufficient command of the German language.

The data of the healthy control groups were drawn from our ongoing database, [12]. All of the participants were examined according to a predefined protocol. This included general health data (maternal age, gravida, para, smoking, CHD in previous children or family members, their own diseases, and maternal intake of medication) through a standardized questionnaire, or the so-called maternal booklet, after inclusion in the study. To assess the level of physical activity before and during pregnancy two standardized questionnaires, both in the control and in the study group, were used: the Activity Questionnaire and the International Physical Activity Questionnaire (IPAQ-SF). The standardized activity questionnaire included: PA before and during pregnancy, and the frequency and type of PA. The different types of activities performed by the respondents were classified into four groups based on a previous observational study: endurance sports, athletic sports, combined sports and light sports [12]. Table 1 displays the different types of sports grouped into the respective column.

A third questionnaire was applied to assess the relevant sociodemographic information of all participants. 

On the basis of the physical activity compendium, the respective metabolic equivalent (MET) can be determined for each of the listed types of sports [13]. With the formula (MET = 3.5 mL O_2_/kg·min or ~1 kcal/kg·h), it is possible to calculate the level of oxygen the body consumes at rest. It can be classified as light (<3.0 METs), moderate (3.0–6.0 METs) and intensive (6.0 METs). Independent of an individual’s physical characteristics, load tolerance, functionality and exercise capacity can be measured in an efficient and standardized way [13]. The individual MET value was calculated for each participant by summing up the MET values for each type of sport she engaged in. 

The questionnaire analysis was conducted with Microsoft Excel and the statistical analysis software RStudio^®^ Version 1.4.1103. To check the data for normal distribution, the Shapiro–Wilk test was performed. The Kruskal–Wallis test was executed to detect differences between the groups. For comparisons between only two groups, the Mann–Whitney U Test was applied. The statistical significance level was set to *p* = 0.05 for all analyses. A logistic regression analysis was used to test whether the predictor variables (maternal age, number of pregnancies, number of abortions, and smoking) have an effect on the outcome variables (physical activity before and during pregnancy, ART, and the fetal diagnosis of CHD).

## 3. Results

In total, 158 pregnant women (ART, CHD and healthy) were included in the study.

### 3.1. Sociodemographic Data

The sociodemographic data of the participants are presented in Table 2.

### 3.2. Activity Questionnaire

The activity questionnaire was used to assess the mothers’ level of physical activity before and during pregnancy. Table 3 presents the subjects’ answers, organized according to the respective groups.

**Figure 1:**
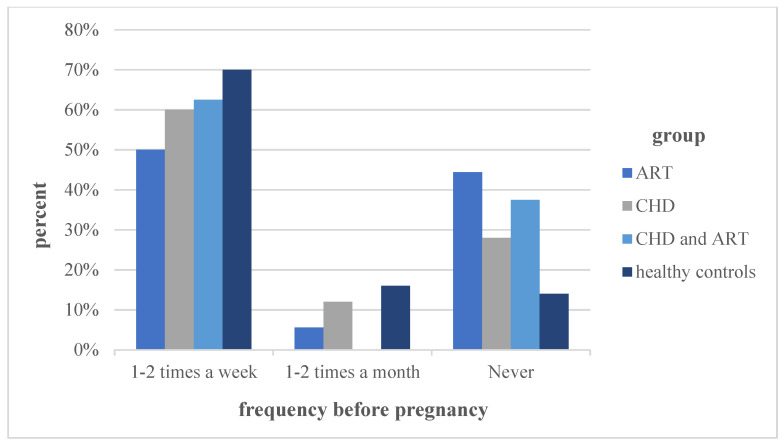
Physical activity frequency before pregnancy. Note: ART = assisted reproduction technologies; CHD = congenital heart defects.

Before pregnancy, the most physical activity was performed by the group of “healthy controls”, followed by the groups “CHD”, “ART and CHD’’. A significant difference was detected between the groups “ART” and “healthy controls” (*p* = 0.014). The frequency of being physically active before pregnancy, classified by times per week/month, did not show any significant differences between the groups. The different frequencies of performing physical activity before pregnancy are graphically illustrated in Figure 1

As described before and illustrated in Table 1, the types of activities were classified as endurance, athletic, combined, and light sports. A significant difference was detected between the groups “ART” (33.3%) and “healthy controls” (83.7%; *p* = 0.032) in the endurance sports category. In the other categories, such as combined sports and lights sports, no significant differences were detected.

**Figure 2:**
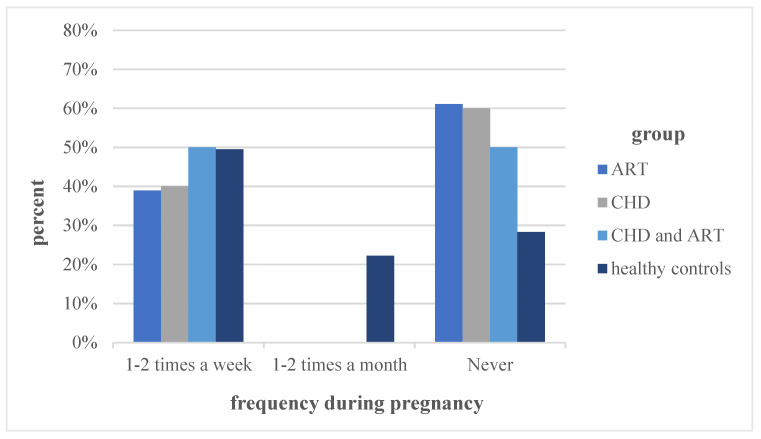
Physical activity frequency during pregnancy. Note: For abbreviations, see Figure 1.

In the overall physical activity during pregnancy, the groups “ART” (44.4%) and “healthy controls” (69%) differed significantly (*p* = 0.029). The different frequencies of performing physical activity during pregnancy are graphically illustrated in Figure 2. The group differences did not show statistical significance.

In general, the level of physical activity in all of the groups was significantly lower during pregnancy compared to before (*p* < 0.001). Figure 1 and Figure 2, together with the statistical analysis, illustrate a significant decrease in the frequency of physical activity in all of the groups during pregnancy compared to before pregnancy (*p* < 0.001). While the number of participants engaging in endurance, athletic and combined sports decreased during pregnancy, the participation in light sports increased significantly in all of the study groups (*p* = 0.002).

A logistic regression was conducted to check whether the predictor variables of maternal age, number of pregnancies, number of abortions and smoking had an effect on the outcome variables of physical activity before and during pregnancy, ART, and the fetal diagnosis of CHD. No significant effect could be detected between the predictor variables and ART. The number of pregnancies (ß = −0.93; *p* = 0.005; 95%CI 2.03; 0.75; OR = 0.4) and maternal age (ß = 0.22; *p* < 0.001; 95%CI 1.13; 1.41; OR = 1.25) were significant predictors of fetal diagnosis of CHD. Similarly, the number of pregnancies (ß = 0.73; *p* = 0.02; 95%CI 1.12; 3.87; OR = 2.07) and maternal age (ß = −0.13; *p* = 0.012; 95%CI 0.79; 9.69; OR = 0.88) were significant predictors of physical activity before pregnancy. The number of previous pregnancies was a significant predictor of physical activity during pregnancy (ß = 0.85; *p* = 0.003; 95%CI 1.36; 4.23; OR = 2.35).

### 3.3. International Physical Activity Questionnaire (IPAQ)

The results of the IPAQ are presented in Table 4.

**Figure 3:**
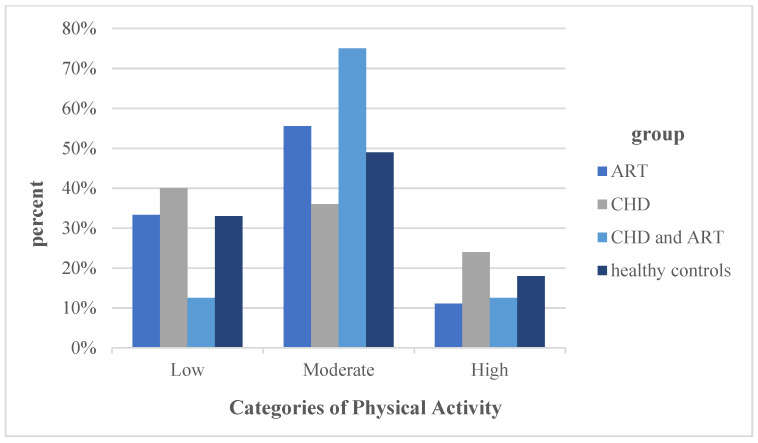
Categorization into physical activity levels. Note: For abbreviations, see Figure 1.

For each group, the median values and interquartile ranges were calculated for walking, moderate-intensity activities, vigorous-intensity activities and a combined total physical activity score. These continuous scores are indicated in MET-minutes/week. Significant differences were detected between the groups “CHD and ART” (*p* = 0.022) as well as between the groups “ART” and “healthy controls” (*p* = 0.009). Based on the duration and intensity of being physically active, the respondents were allocated to one of the three categorical scores: low, moderate or high levels of physical activity. Subsequently, the percentages of the individual groups were determined for each level of physical activity, which are illustrated in Figure 3. No statistical significance could be detected between the groups. Comparing the medians of the sitting time in hours per week between the four groups, the group “CHD and ART” spent the most time sitting, followed by the “healthy controls”. None of the group differences showed statistical significance.

## 4. Discussion

The study showed that there was a significant reduction in physical activity during pregnancy in healthy, as well as risk, pregnancies. The results also showed that only about a quarter (26.49%) of all pregnant women who participated in the study achieved the WHO recommendations. The “healthy controls” engaged in the most overall physical activity during pregnancy. The difference between the “healthy controls” and the group after “ART” was significant, and showed that women after ART perform less physical activity during pregnancy. Despite an excessive search in various scientific databases, no research could be identified assessing the influence of ART on the level of physical activity during pregnancy by comparing pregnancies after ART and natural pregnancies. However, one study investigated the physical activity behavior before and during ART among women experiencing infertility. A significant decrease in physical activity could be detected in women after ART. According to the findings, more than 80% of women feared that not cutting down on physical activity might negatively affect the outcome and decrease the chance of pregnancy [14]. A qualitative study explored the experiences of Taiwanese mothers after a successful ART treatment. The women interviewed reported less physical activity overall, to the extent of even choosing to stay at home during pregnancy [15]. Even if no direct comparison between women who became pregnant in a natural way and women after ART was conducted, the findings confirmed that the overall physical activity is reduced in the group after ART.

The IPAQ revealed that the group after ART spent the least time walking; similarly to the Activity Questionnaire, a significant difference was detected between this group and the “healthy controls”. Therefore, it can be concluded that the group after ART influenced the maternal level of walking negatively compared to a naturally conceived pregnancy. 

Based on the frequency of performing physical activity during pregnancy, it was demonstrated that the “healthy controls” are more often physically active than the other groups. The METs supported this finding, since the “healthy controls" displayed the highest value. The differences in the frequencies before compared to during pregnancy were significant. While the number of participants engaging in endurance, athletic and combined sports decreased during pregnancy, the participation in light sports increased significantly in all of the study groups.

The detected significant difference between physical activity behavior before and during pregnancy is in line with previous research. Researchers assessed physical exercise among healthy pregnant women in Germany, and identified a significant decrease compared to the pre-pregnancy period. In addition, other researchers confirm that pregnant women prefer light or sedentary activities rather than moderate intensity activities [16,17].

Statistical significance was observed in several relationships. An increase in maternal age increased the odds for a fetal diagnosis of CHD. It was also determined, that an increase in maternal age decreased the odds for being physically active before pregnancy. If an additional previous pregnancy had been experienced, the odds for being active before and during pregnancy increased. Similarly to this study, a large Danish study identified high maternal age as an influencing factor [18]. The study showed that an additional previous pregnancy was associated with an increase in the physical activity level before and during pregnancy. Another study was conducted in the USA. The results indicated that a maternal age of 35 increased the prevalence of certain CHD phenotypes, for instance, laterality defects, all conotruncal defects, VSD and ASD [19]. In contrast, a study conducted in the UK could not find an association between the maternal age at delivery and the prevalence of CHD [20]. The systematic review and meta-analysis by Jonau-Zoulovits et al. did not only focus on the mother’s age but also the father’s. The researchers found that advanced parental age increased the odds for developing CHD by 16% [21]. Due to the contradictory findings in existing literature, further research assessing the influence of maternal age on CHD is necessary.

The study revealed that the women in the group “CHD and ART” did not reach the WHO recommendations during pregnancy. Only 12.5% were physically active, as is recommended, in the group of pregnant women after ART and who received the diagnosis of fetal CHD. Again, this finding highlights the need for interventions aiming to increase physical activity among these women.

Previous studies have identified several potential reasons explaining the physical inactivity during pregnancy. It is assumed that barriers hinder the target group from being sufficiently active. For instance, pregnant women often report pregnancy-related symptoms, such as lumbopelvic pain, anxiety and depression, and gestational weight gain, limiting their ability to being physically active [22,23,24]. Another explanation is a busy time schedule due to children, work or family responsibilities, which decreases the amount of time available for engaging in physical activity [10,25]. Another major barrier is the lack of information and knowledge about safe exercise during pregnancy, as well as the associated benefits for the mother and child [26]. In contrast, some women who are aware of the benefits consider rest and relaxation to be more relevant than movement during pregnancy [27]. Women after ART were worried that physical activity might negatively affect the outcome and decrease the chances of achieving pregnancy [14]. Despite the advice of continuing physical activity after embryo transfer, many women after ART portray bed rest as more appropriate, and therefore reduce their activity level [28]. 

The WHO recommendations do not include information on physical activity for pregnancies in the target groups. These populations are presumably unsure of whether the guidelines are equally applicable to them and can be utilized as an orientation.

Prevention and intervention strategies also need to target the group of pregnant women more specifically. A systematic review analyzed the effectiveness of physical activity interventions among pregnant women; it concluded that physical activity interventions positively influenced the level of exercise during pregnancy [29]. Miquelutti et al. evaluated the effectiveness and safety of a birth preparation program aiming to reduce lumbopelvic pain, urinary incontinence and anxiety, and increase exercise, during pregnancy. The intervention program included physical exercises, educational activities and instructions for individual exercises at home. While the intervention group increased their level of physical activity, the control group showed a decrease [30]. It has to be advised that holistic interventions specifically designed for pregnant women have to be incorporated into public health programs. Since physical activity decreases as pregnancy advances, interventions need to be developed that accompany women during the whole pregnancy [18,31]. Such programs should be initiated at the planning stage of pregnancy and continue even after delivery. According to Chan and colleagues [29] a holistic concept should comprise several components, including mandatory physical activity classes, physical activity education, and the provision of appropriate exercises that are safe for pregnant women. A combination of dietary, physical activity and behavioral knowledge and methods is optimal for increasing physical activity levels among pregnant women [32,33]. The duration and intensity of the exercise classes should be based on the WHO guidelines on physical activity during pregnancy, and take pregnancy-related symptoms into account. Advice on how easily incorporate physical activity into the daily schedule should be provided [29]. Supervised group sessions could reduce the perceived barrier of lack of motivation and therefore increase participation [34].

For the specific target groups, the general programs need to be extended. This need is further supported by the findings of this study. As previously discussed, research has proven that physical activity before and during the ART treatment does not influence the outcomes negatively [35,36]. In addition, maternal exercise decreases the fetal risk for several diseases, such as congenital abnormalities [4,37]. Moreover, a specific focus has to lie on women with a high maternal age; furthermore, the present research has identified that an increase in maternal age increases the likelihood of CHD and is also associated with a decrease in the level of physical activity before pregnancy. Health care programs and health care providers should educate the mentioned target groups about the benefits of physical activity during pregnancy to motivate them to begin or continue exercising.

The limitations of the study are that the three groups “CHD”, “CHD and ART” and “ART”, have a small sample size. Due to the study’s connection to physical activity, it cannot be ruled out that a selection bias might have occurred. The cross-sectional study design does not allow us to establish and derive causal relationships. The survey only addressed the level of physical activity, but did not examine the underlying reasons that explain low levels of exercise, for example. Overall, since the listed shortcomings might limit the findings’ validity and generalizability, they need to be interpreted with caution.

## 5. Conclusions

The level of physical activity during pregnancy is influenced by ART but no clear association to the diagnosis of fetal CHD, or both CHD and ART, could be detected. Therefore, it can be stated that, in this study, women after ART showed a reduced level of physical activity during pregnancy compared to women who became pregnant in a natural way. The WHO recommendations for physical activity could not be achieved, especially in these groups. Additionally, a difference in the physical activity behavior before and during pregnancy was detected. Less and lighter physical activity was observed in all of the groups during pregnancy. An influence of individual-related parameters, namely maternal age and number of previous pregnancies, on physical activity and CHD was detected. Since even after an extensive literature search, no studies assessing the influence of ART and CHD on the maternal level of physical activity could be detected, a relevant research gap was identified. Further studies for specific target groups are needed, to gain knowledge related to high-risk pregnancies. When planning and conducting future research, the aforementioned limitations and shortcomings have to be taken into account. 

## Figures and Tables

**Table 1:** Classification of the different sports.

Athletic Sports	Endurance Sports	Light Sports	Combined Sports
EMS	Aerobic	Aqua gymnastics	Badminton
Fitness	Endurance	Gymnastics	Ballet
Defined back training	Speed ice-skating	Pilates	CrossFit
Climbing	Jogging	Back gym	Figure skating
Karate	Inline skating	Walking	Soccer
	Cross-country skiing	Yoga	Handball
	Treadmill	Bow shooting	Horseback riding
	Running	Muscle building	Skiing
	Biking	Stretching	Squash
	Rowing		Dancing
	Swimming		Table tennis
	Spinning		Volleyball
	Hiking		Wakeboard
	Zumba		

Note. Table adapted from Sitzberger et al. 2020 [12]. EMS: electrical muscle stimulation.

**Table 2:** Sociodemographic data of the four groups.

	ART (A)	CHD (B)	CHD and ART (C)	Healthy Controls (D)	*p*-Value	*Post Hoc*
Number (%)	18 (11.4%)	25 (15.8%)	8 (5.1%)	107 (67.7%)	/	
Maternal age (Mean ± SD in years)	35.78 ± 4.31	34.28 ± 4.89	33 ± 2.88	32.96 ± 4.27	**0.002**	***B–C*** ***C–D***
Number of pregnancies (Mean ± SD)	1.33 ± 0.77	2.44 ± 1.85	1.63 ± 0.52	2.08 ± 0.94	**0.002**	***A–B*** ***A–D***
Number of abortions (Mean ± SD)	0.17 ± 0.38	0.72 ± 1.54	0.25 ± 0.46	0.67 ± 0.74	**0.008**	***A–D***
Number of previous children (Mean ± SD)	0.17 ± 0.51	0.72 ± 0.89	0.38 ± 0.52	0.41 ± 0.58	0.093	*/*
Smoking (%)	11.1	4	0	3.7	0.400	*/*
CHD in previous children (%)	/	5.6	33.3	/	0.388	*/*
CHD in family members (%)	/	16	25	/	0.572	*/*
Maternal Diseases (%)	/	8	25	/	0.207	*/*

Note. Results are based on Kruskal–Wallis or Mann–Whitney U tests due to not normally distributed data. Significance level for upper case letters (A, B, C, D): 0.05. The bold values indicate a significant difference. Tests are adjusted for all pairwise comparisons within a row using the Bonferroni correction.

**Table 3:** Data assessed by the physical activity questionnaires of the four groups.

		ART (A)	CHD (B)	CHD and ART (C)	Healthy Controls (D)	*p*-Value	*Post Hoc*
PA before pregnancy (%)		55.6	72	62.5	86	**0.013**	***A–D***
Frequency of PA before pregnancy (%)	1–2x/week: 1–2 x/month: Never:	50 5.6 44.4	60 12 28	62.5 0 37.5	70 16 14	0.163	*/*
Types of Activity before Pregnancy (%)	E: A: C: L:	33.3 5.6 11.1 33.3	48 8 20 40	50 25 0 25	83.7 34.9 19.8 24.4	**0.021** 0.063 0.508 0.176	***A–D*** */* */* */*
PA during Pregnancy (%)		44.4	40	50	69	**0.006**	***A–D***
Frequency of PA during Pregnancy (%)	1–2x/week: 1–2 w/month: Never:	38.9 0 61.1	40 0 60	50 0 50	49.5 22.2 28.3	0.142	*/*
Types of Activity during Pregnancy (%)	E: A: C: L:	16.7 0 0 38.9	24 4 8 28	25 0 0 37.5	63.4 18.3 8.5 56.3	0.092 0.165 0.570 0.70	*/* */* */* */*
MET value (Mean ± SD)		4.11 ± 6.5	3.9 ± 5.81	4.38 ± 7.58	4.73 ± 2.55	0.284	*/*

Note. PA = physical activity; E *=* endurance sports, A = athletic sports, C = combined sports, L = light sports. Results are based on Kruskal–Wallis or Mann–Whitney U tests due to not normally distributed data. Significance level for upper case letters (A, B, C, D): 0.05. The bold values indicate a significant difference. Tests are adjusted for all pairwise comparisons within a row using the Bonferroni correction.

**Table 4:** Data assessed by the IPAQ of the four groups.

		ART	CHD	CHD and ART	Healthy Controls	*p*-Value
Walking MET-minutes/week (Median/IQR)		321.75/532.13	1188/1386	1386/1794.38	957/990	**0.005**
Moderate MET-minutes/week (Median/IQR)		510/1350	0/120	0/150	170/720	0.013
Vigorous MET-minutes/week (Median/IQR)		0/270	0/0	0/0	0/0	0.833
Total physical activity MET-minutes/week (Median/IQR)		1749/1455.75	1782/1011	1682.25/2452.5	1386/1828.1	0.888
Categorical Score (%)	Low: Moderate: High:	33.33 55.56 11.11	40 36 24	12.5 75 12.5	33 49 18	0.881
Sitting time in hours/day (Median/IQR)		4.5/6.13	4.5/4	5.5/4.75	5/4.13	0.999

Note. MET = metabolic equivalent; IQR = interquartile range. Results are based on Kruskal–Wallis or Mann–Whitney U tests due to not normally distributed data. The bold values indicate a significant difference. Tests are adjusted for all pairwise comparisons within a row using the Bonferroni correction.
